# The First CRISPR-Based Therapeutic (SL_1.52) for African Swine Fever Is Effective in Swine

**DOI:** 10.3390/v17111504

**Published:** 2025-11-14

**Authors:** Naveen Verma, Alison O’Mahony, Roky Mohammad, Dylan Keiser, Craig W. Mosman, Deric Holden, Kristin Starr, Jared Bauer, Bradley Bauer, Roypim Suntisukwattana, Waranya Atthaapa, Angkana Tantituvanont, Dachrit Nilubol, Douglas P. Gladue

**Affiliations:** 1 Seek Labs, 350 W 800 N, Salt Lake City, UT 84103, USA; naveen@seeklabs.com (N.V.); alison@seeklabs.com (A.O.); roky@seeklabs.com (R.M.); craig@seeklabs.com (C.W.M.); deric@seeklabs.com (D.H.); kristin@seeklabs.com (K.S.); jared@seeklabs.com (J.B.); brad@seeklabs.com (B.B.); 2 Swine Viral Evolution and Vaccine Development Research Unit, Department of Veterinary Microbiology, Faculty of Veterinary Science, Chulalongkorn University, Henry Dunant Road, Pathumwan, Bangkok 10330, Thailand; 3 Department of Pharmaceutic and Industrial Pharmacies, Faculty of Pharmaceutical Sciences, Chulalongkorn University, Bangkok 10330, Thailand

**Keywords:** ASFV, ASF, African swine fever virus, CRISPR, Cas9, therapeutic, immunity

## Abstract

African swine fever virus (ASFV) is a high-consequence pathogen that causes African swine fever (ASF), for which mortality rates can reach 90–100%, with death typically occurring within 14 days. ASF is currently a highly contagious pandemic disease responsible for extensive losses in pig production in multiple affected countries suffering from extended outbreaks. While a limited number of vaccines to prevent ASF are in use in south-east Asia, vaccines are not widely available, are only effective against highly homologous strains of ASFV, and must be used prior to an outbreak on a farm. Currently, there is no treatment for ASF and culling affected farms is the only response to outbreaks on farms to try and prevent spreading. CRISPR/Cas systems evolved as an adaptive immune response in bacteria and archaea that function by cleaving and disrupting the genomes of invading bacteriophage pathogens. CRISPR technology has since been leveraged into an array of endonuclease-based systems used for nucleic acid detection, targeting, genomic cleavage, and gene editing, making them particularly well-suited for development as sequence-specific therapeutic modalities. The programmability of CRISPR-based therapeutics offers a compelling new way to rapidly and specifically target pathogenic viral genomes simply by using different targeting guide RNAs (gRNA) as an adaptable antiviral modality. Here, we demonstrate for the first time a specific CRISPR/Cas9 multiplexed gRNA system that targets the African swine fever viral genome, resulting in sequence-specific cleavage, leading to the reduction in the viral load in infected animals, and subsequent recovery from an otherwise lethal dose of ASFV. Moreover, animals that recovered had protective immunity to subsequent homologous ASFV infection.

## 1. Introduction

Emerging and re-emerging viral diseases increasingly threaten both human and animal health. Traditional vaccine and drug development pipelines depend on prior pathogen characterization, leaving significant gaps when new viral outbreaks occur. African swine fever virus (ASFV) is a double-stranded DNA virus that causes African swine fever (ASF), a highly contagious and often fatal disease in domestic and wild swine populations. ASFV infections can cause devastating economic losses both for the affected farms as well as in outbreak regions that rely on exporting swine or swine products. ASFV-infected animals present the disease in a variety of clinical forms depending on the acting virus isolate and the characteristics of the infected host [1]. Since its discovery in 1921 [2], ASF has been mostly restricted to the sub-Saharan African region; however, in 2007, the disease quickly spread across Eurasia after a single disease introduction in the republic of Georgia [3]. In 2021, for the first time since 1980, ASFV was introduced into the Dominican Republic and Haiti, likely through infected meat transported by plane from Mediterranean countries, and resulted in widespread infection on the island of Hispaniola [4]. This outbreak caused a severe decline in Haiti’s pig population and meat production levels. During the 6 years of the eradication effort, live pig numbers dropped by 71% and meat processing fell by 43%. The 1980 outbreak of ASFV was only controlled by massive culling of all swine on the island and it has taken more than 25 years for swine population to recover [5]. Currently, the strain of ASFV on the island of Hispaniola resembles the outbreak strain in Eurasia [4], demonstrating the first spread of the Eurasian outbreak strain to the Western Hemisphere. This current ASF pandemic has severely affected pig production—causing food shortages worldwide—and current procedures for control and prevention continue to rely on diagnostics, highly restrictive pig movement, and culling of infected animals. Unfortunately, this approach has been largely ineffective in controlling the spread of the disease, and new countries that had been free of ASFV are now reporting outbreaks every year since the 2007 introduction in the Republic of Georgia.

ASFV is a structurally complex virus, harboring a large double-stranded DNA genome of approximately 180–190 kilobase pairs encoding more than 150 genes [6]. The correlates for immune protection are unknown, despite the commercialization of some vaccines in Vietnam and the predicted structures of all ASFV proteins being publicly available [7]. Currently, there is no treatment for ASF, and the only vaccines are recombinant viruses harboring deletions of specific genes that have limited protection against genetically similar ASFV strains, as reviewed in Gladue et al. 2022 [8]. Despite having a large and complex genome, next-generation sequencing (NGS) efforts [9,10,11] have led to a standardized analysis platform [12], allowing reliable sequencing of full ASFV genomes. Recent reclassification of ASFV identified 7 different biotypes [13]; however, the cross-protection within or between biotypes has not yet been widely reported. While some reports have observed biotype 1 and biotype 2 cross-protection [14], new strains have emerged recently in Asia, largely as a consequence of using non-homologous vaccines. The resulting new biotype (1/2) is a hybrid of biotypes 1 and 2 [13] that is escaping biotype 2 vaccines [15]. The emergence of this hybrid 1/2 biotype highlights the urgent need for a universal vaccine or an effective treatment for ASFV that is biotype independent and does not require NGS for new outbreaks.

The use of CRISPR/Cas9 to combat pathogens is not a new idea. These endonuclease systems were first discovered in nature as a prokaryotic defense mechanism to combat invading viruses [16,17,18]. Genetic engineering has allowed researchers to leverage the adaptability of CRISPR/Cas systems, which can be programmed using specific guide-RNAs (gRNAs) to precisely target, cleave, and edit genomes and transcriptomes in mammals [19]. Here, we leverage the programmability of CRISPR/Cas to develop an antiviral that targets the pathogen’s genomic sequence to disrupt viral replication and limit disease. In contrast to traditional vaccine and antiviral development, requiring a comprehensive understanding of viral protein function, mechanism of action and immune impacts, gRNA design requires only the target genomic sequence to cleave in the virus genome. The therapeutic potential of CRISPR has been validated in humans with Casgevy, the first Cas9-based treatment of sickle cell disease, approved by the U.S. Food and Drug Administration (FDA) on 8 December 2023 [20]. Approval for another indication, beta-thalassemia, quickly followed on 16 January 2024. [20]. This one-time treatment is considered curative as it addresses the root cause of the disease and trial patients were reported to be symptom-free for one year or more. Other groups have built on this success to deploy CRISPR systems to fight other mutational and infectious diseases [21,22,23]. In this study, we utilized CRISPR/Cas9 and a multiplex guide approach to target open-reading frame (ORF) *G1211R*, which encodes a DNA polymerase in ASFV reported to be involved in replication of the viral genome [24]. Our CRISPR system programmed with multiplexed gRNAs is specifically designed to target the current pandemic ASFV pathogen, as *G1211R* is highly conserved in all biotype 2 and biotype 1/2 strains. This approach offers the potential for reduced viral burden and full recovery from a lethal dose of ASFV. When treated with a CRISPR/Cas9 system specifically targeting ASFV genes, infected animals can mount an effective immune response to clear the virus and, crucially, develop protective immunity to prevent subsequent infection by a homologous strain of ASFV.

## 2. Materials and Methods

### 2.1. Viruses and Cell Culture

The ASFV pathogenic agent used in this study was prepared from an infected spleen harvested from a swine with severe clinical symptoms that tested positive for ASFV. The viral titer of the spleen homogenate was determined by hemadsorption dose (HAD) using primary cultures of swine macrophages, prepared as previously described [25] and used at a density of 5 × 10^6^ cells per mL. Titer was calculated as previously described using the Reed–Muench method for virus titration calculation [26]. An intramuscular dose of 10^2^ HAD was used for swine infection studies. NGS was performed on the inoculum confirming that the strain used in this study was the pandemic type 2 strain that closely resembles that of other outbreak strains in Eurasia.

HEK293T cells were grown in growth media consisting of Dulbecco’s Modified Eagle’s Medium (DMEM) (ATCC 30-2002, Manassas, VA, USA) supplemented with 10% Fetal Bovine Serum (FBS) (ATCC 30-2020), 1% GlutaMAX (GIBCO 35050061, Thermo Fisher Scientific, Waltham, MA, USA), and 1% PS (Penicillin and Streptomycin) (ATCC, 30-2300, Manassas, VA, USA). Cells were maintained at 37 °C with 5% CO_2_.

### 2.2. Design of the Guide Sequences for ASFV

Guide RNA (gRNA) sequences were designed to target current ASFV strains circulating in the pandemic. gRNAs were then selected based on efficient Cas9 complex formation and cleavage, PAM requirements, and potential off-target effects on host genome [27]. Specifically, in Georgia/2007 Accession # FR682468.2, the ORF for *G1211R* is between nucleotide positions 114,158 and 11,778 on the positive strand of ASFV. The guides were inserted in the CRISPR/Cas9 construct, SL_1.52, and are listed in Table 1. The gRNA1 is targeted toward the N-terminal region of *G1211R* at nucleotide positions 27–46, and gRNA 2 is targeted toward the C-terminal region of *G1211R* at nucleotide positions 1677–1697.

### 2.3. Construction of SL_1.52 and Target Plasmids

Using the pCDNA 3.12 plasmid backbone, CRISPR/Cas9 ASFV targeting plasmid (SL_1.52) was constructed using a CMV enhancer and promoter to express Cas9 followed by sequences encoding a SV40 nuclear localization signal (NLS), a *Thosea asigna* virus 2A (T2A) self-cleavable linker [28], followed by a Cop-GFP sequence. SL_1.52 also contains a multiplex gRNA cassette consisting of two guides, each under U6 promoters, such that each gRNA spacer sequence, complimentary to ASFV target sequences (shown in Table 1), is fused with a Cas9 TracrRNA/Direct repeat sequence [29].

The *G1211R* gene or a control non-targeted *P1192R* gene from ASFV-Georgia/2007 were cloned into the pCDNA 3.12 backbone followed by the T2A linker and red fluorescence protein (RFP). The two plasmids constructed were pcDNA-*G1211R*-T2A-RFP (Targeting; T) and pcDNA-*P1192R*-T2A-RFP (Non-targeting; NT).

Plasmids were synthesized and propagated with the removal of endotoxins by GenScript (Piscataway, NJ, USA).

### 2.4. Biochemical Validation of Guide Sequences

First, single guide RNA (sgRNA) was synthesized in vitro using EnGen^®^ sgRNA Synthesis Kit (E3322V) from New England Biolabs (NEB, Ipswich, MA, USA). Briefly, the EnGen 2X sgRNA Reaction Mix, along with 0.1 M DTT, target-specific oligo, and enzyme mix was incubated at 37 °C for 30 min. For DNase treatment, the volume was adjusted to 50 μL by adding 30 μL of nuclease-free water, followed by the addition of 2 μL of DNase I (RNase-free) and incubating at 37 °C for 15 additional minutes. The RNA purification kit (NEB, T2040S) was used to purify the sgRNA. Second, for in vitro digestion, the reaction was assembled at room temperature in the following order: 20 μL of nuclease-free water, 3 μL of 10× NEBuffer r3.1 Reaction Buffer, 3 μL of 300 nM sgRNA (to achieve a final concentration of 30 nM), and 1 μL of 1 μM Cas9 (NEB M0386S), resulting in a final concentration of approximately 30 nM. Reaction mixture was pre-incubated for 10 min at 25 °C. Then, 3 μL of 30 nM substrate DNA (pcDNA *G1211R*-T2A-RFP) was added to reach a final concentration of 3 nM. The mixture was mixed thoroughly and pulse-spun in a microfuge, followed by incubation at 37 °C for 30 min. Subsequently, 1 μL of Proteinase K was added to each sample, thoroughly mixed, and pulse-spun in a microfuge, followed by incubation at room temperature for 10 min. The reaction was resolved on 0.6% agarose gel, stained with Gel Green (Biotium, 41005, Fremont, CA, USA), and imaged with UVP ChemStudio (Analytic Jena, Jena, Germany).

### 2.5. In Vitro Cleavage of ASFV Target Sequences

HEK293T cells were seeded at 1.25 × 10^5^ cells per well in 24-well plates (VWR, cat #10861-700, Radnor, PA, USA) using 0.5 mL of DMEM complete growth medium the day before transfection to obtain 50–80% confluency of actively growing cells on the day of transfection. Transfection complexes were prepared by mixing an equimolar aliquot of SL_1.52 and ASFV target gene plasmid totaling 250 ng of DNA, along with 0.5 µL TransfeX Transfection Reagent (ATCC, cat# ACS-4005, Manassas, VA, USA) reagent in 50 µL Opti-MEM (Thermo Fisher Scientific, cat# 51985091, Waltham, MA, USA) and incubating for 15 min. The complexes were added dropwise to the cells, followed by gentle rocking to evenly distribute the mixture. Cells were incubated at 37 °C with 5% CO_2_ for 48 h, where RNA was extracted using Monarch^®^ Total RNA Miniprep Kit (NEB, cat #T2010S) according to manufacturer instructions. 50 ng of Total RNA from each sample was amplified using qRT-PCR Brilliant III Probe Master Mix (Agilent, cat #600884, Santa Clara, CA, USA) in AriaMx Real-time PCR System (Agilent) in a 20 µL reaction mix containing 10 µL of the 2× master mix, 0.250 µM of each forward and reverse primer, and the fluorogenic probe, and nuclease-free water to adjust the final volume under the following conditions: RNA to cDNA conversion at 65 °C for 10 min, followed by an initial denaturation at 95 °C for 3 min, then 40 cycles of denaturation at 95 °C for 5–10 s, and amplification at 60 °C for 30 s. Relative fold change in the expression of target and non-target genes was determined by Livak method. Relative quantification of the genes was determined by comparing the gene expression in the samples co-transfected with pUC19 or SL_1.52. Each experiment consisted of three technical replicates and three biological replicates were carried out to determine statistical significance.

### 2.6. Lipid Nano-Particle (LNP) Generation

LNPs were generated using SL_1.52 plasmid and a 4-part lipid mixture. Plasmid DNA solution was prepared at 100 ng/µL concentration in 50 mM sodium acetate pH 4.5 acidic solution and then filtered through a 0.45 µm PES membrane filter for sterility and to remove any particulates. 4-part lipid mixture was prepared using SM-102, 1,2-DSPC, Cholesterol, and DMG-mPEG (2000) at 50:10:38.5:1.5% molar ratio in ethanol. Each of the above components were individually dissolved in 100% ethanol at 100 mg/mL SM-102, 25 mg/mL 1,2-DSPC, 5 mg/mL Cholesterol, and 1 mg/mL DMG-mPEG (2000) to obtain separate clear solutions. Then, solutions were mixed to obtain the above molar ratios in the 4-part mixture as previously described [30]. Specifically, 1 mL of the 4-part lipid contained 35 µL of 100 mg/mL SM-102, 32 µL of 25 mg/mL 1,2-DSPC, 296 µL of 5 mg/mL Cholesterol, and 378 µL of 1 mg/mL DMG-mPEG (2000), and larger volumes were prepared by scaling up proportionally. The 4-part lipid was filtered through a 0.45 µm filter for sterility and to remove any undissolved particulate matter. LNPs were generated on a PreciGenome Nanoparticle Generator using the CHP-MIX-3 microfluidic mixer chip (PreciGenome, San Jose, CA, USA). A ratio of 3:1 of the aqueous phase (plasmid DNA solution) to organic phase (4-part lipid mixture) was used and the aqueous to organic flow rate ratio (FRR) was also set to 3:1.

### 2.7. Evaluation of SL_1.52 as a Thereputic in Swine

The in vivo evaluation of SL_1.52 as an anti-viral therapeutic modality was performed in swine that were housed in separate pens in an outdoor farm. 5 days prior to initiating the study, swine were tested for ASFV antibodies using ID Screen^®^ African Swine Fever Competition (ID.Vet, Montpellier, France), following the manufacturer’s instructions and using the recommended score of 40% as a cutoff for background or nonspecific ASFV Ab levels. All swine tested negative for ASFV antibodies and were randomly distributed between two groups. Swine in both groups were inoculated intramuscularly (IM) with 10^2^ ASFV/Thailand/2023 preparation. On days 5 and 6 post-infection, pigs in the treated group were given SL_1.52 encapsulated in LNPs as described in Section 2.6. The presence of ASF-related clinical signs, such as anorexia, depression, fever, purple skin discoloration, staggering gait, diarrhea, and cough, were monitored daily. Body temperatures were recorded daily throughout the study; additionally, ambient temperatures were also tracked to assess if a subject’s temperature was related to high ambient temperatures typically observed in outdoor farm facilities. Blood samples were scheduled to be collected every 5 days post inoculation (pi). Viral titers in blood were determined using real-time PCR using VetMAX African Swine Fever Virus Detection Kit (Thermo Fisher Scientific, Cat # A28809, Waltham, MA, USA) as per the manufacturer’s instructions.

## 3. Results

### 3.1. SL_1.52 Construction and Design of Guides and Cas Molecules

We utilized a single expression plasmid to deliver both the Cas9 enzyme and a multiplex cassette encoding two gRNAs. gRNA1 and gRNA2 are listed in Table 1 and were designed to target the ASFV ORF for *G1211R* between nucleotide positions 114,158 and 11,778 in the Georgia/2007 genome. Specifically, gRNA1 targets the N-terminus of the ORF at positions 27–46, and gRNA2 targets the C-terminus of the ORF at positions 1677–1697. The distance between the gRNAs was set to ensure there was no competition for cleavage from either of the gRNAs. *G1211R* encodes a viral DNA polymerase, which initiates ASFV replication and is a highly conserved viral protein [24]. Both target sequences in the ASFV genome are conserved in all biotype 1 and biotype 1/2 strains of ASFV currently causing outbreaks outside of Africa. Although ASFV replication primarily occurs in viral factories formed in the cytoplasm following disruption of nuclear organization, an early brief replication phase occurs in the nucleus [31,32]. For this reason, a Cas9 attached to a SV40 nuclear localization signal was used to facilitate binding and cleavage of the viral genome in the early replication phase in the nucleus and later in the cytoplasm after nuclear membrane disruption, prior to the formation of viral factories.

### 3.2. Evaluation of SL_1.52 Cleavage In Vitro

**Figure 1:**
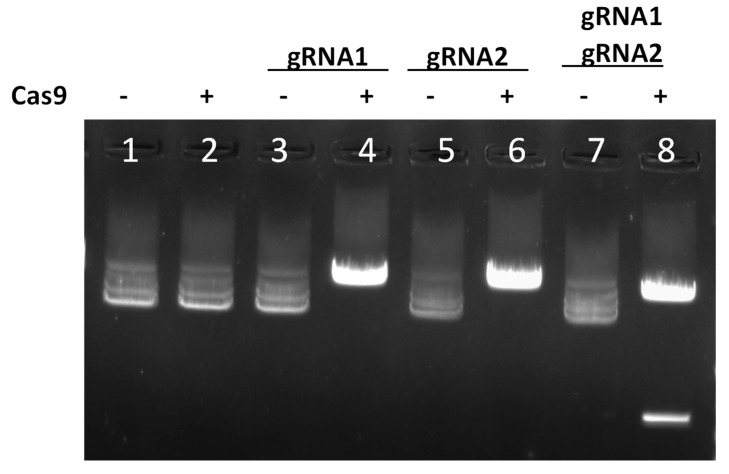
RNPs consisting of Cas9 complexed with the indicated guides were incubated with G12211R target plasmid and cleavage bands visualized on an agarose gel. Lane 1 contains supercoiled uncut plasmid only and lanes 2, 3, 5, and 7 contain uncut plasmid incubated with either Cas9 or gRNA(s) only. Lanes 4, 6, and 8 show cut plasmid bands following incubation with Cas9 RNP complexed with the indicated gRNA(s).

To test if the designed gRNAs could effectively cleave target DNA in a biochemical assay, we combined each gRNA with a Cas9 protein to form a ribonucleoprotein (RNP) complex and incubated it with the *G1211R*-RFP expression vector as described in the materials and methods. When we incubated this RNP complexed with either gRNA1 or gRNA2 targeting G1211R, we observed linearization of the supercoiled plasmid vector, indicating a single cleavage event within the *G1211R* encoding sequence. Additionally, when we incubated Cas9 protein complexed with gRNA1 plus gRNA2, we observed cleavage events that yielded two DNA fragments of approximately 1651 bp and 7747 bp as expected from the predicted dual cleavage achieved with gRNA1 and gRNA2 design (Figure 1).

### 3.3. Evaluation of SL_1.52 Knockdown of Target Genes in Mammalian Cell Culture

**Figure 2:**
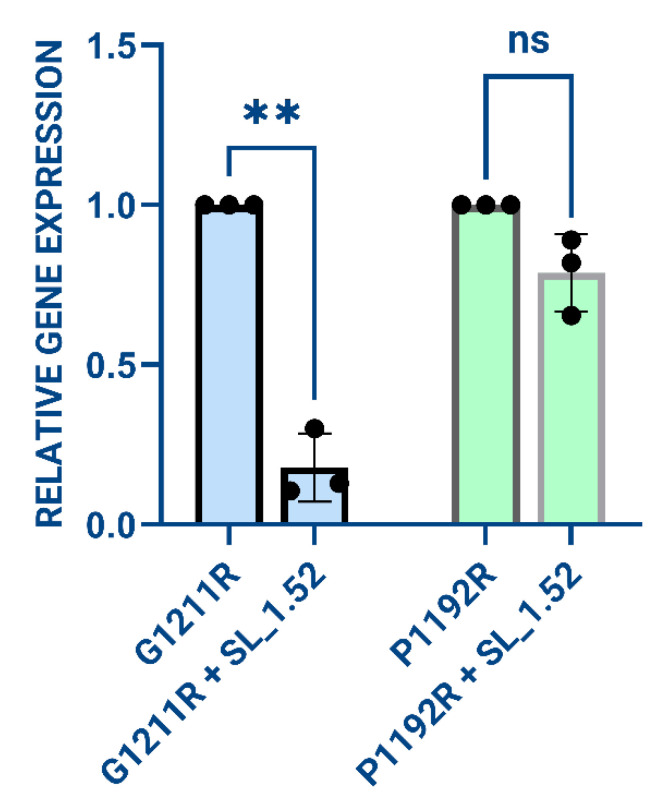
The relative gene expression of *G1211R* (targeted) and *P1192R* (non-targeted) when transfected with or without SL_1.52 was determined by qPCR for the indicated gene and relative gene expression was determined in the absence or presence of SL_1.52, graphed in GraphPad Prism (Version 10.2.1), and statistical significance was determined by non-parametric Student’s *t* test. ** = *p*-value ≤ 0.01; ns = non-significant.

The SL_1.52 CRISPR construct and expression vectors encoding either targeted (T) *G1211R* or non-targeted *P1192R* (NT) genes were co-transfected into HEK293T cells, as described in the materials and methods. After 48 h, DNA was extracted and RT-qPCR for *G1211R* or *P1192R* transcripts were performed to measure knockdown of relative gene expression (RGE). Only cells that contained both SL_1.52 and *G1211R* T sequence showed RGE knockdown with the transfected target sequence. No cleavage and therefore no target knockdown was observed in cells transfected with SL_1.52 plus the control *P1192R* NT plasmid (Figure 2).

### 3.4. Evaluation of ASFV Virulence in Pigs Treated with ASFV SL_1.52 Targeting ASFV

**Figure 3:**
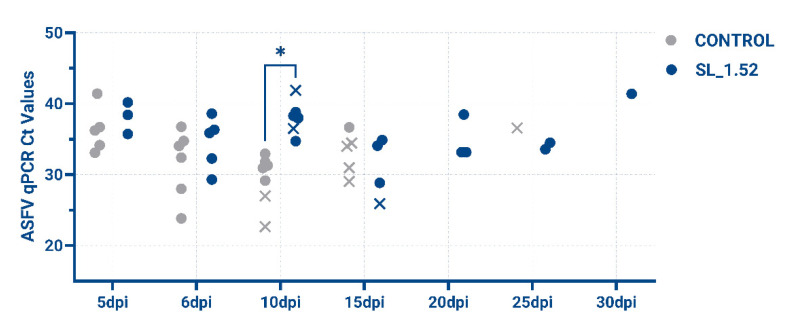
qPCR values of individual pigs during the course of the experiment. In grey are untreated controls, in blue pigs are treated, and blue circles represent surviving animals. A Mann–Whitney U Test with FDR correction using the two-stage step-up method of Benjamini, Krieger, and Yekutieli (BKY) with an FDR set to 5% on the 2^^−Ct^ values of the viral loads gave a q value marked with an * of 0.0018.

Commercially bred swine between 6–22kg were confirmed negative for ASFV infection and ASFV antibodies (ID.Vet) to rule out any previous exposure to ASFV prior to this study. Pigs were randomly distributed between groups and housed in separate pens. The effectiveness of SL_1.52 treatment compared to non-treated groups was evaluated in 9–16 kg pigs. Groups of pigs (*n* = 7) were intramuscularly (IM) inoculated with 10^2^ HAD_50_ of ASFV/Thailand/2023. At 5 dpi (days post infection) and 6 dpi, all pigs in the treatment cohort received a dose of SL_1.52 encapsulated by lipid nanoparticles by IM injection at six distinct sites: on each side of the upper back, hind quarters, and leg muscles. The presence of clinical signs associated with ASF (such as anorexia, depression, fever, purple skin discoloration, staggering gait, diarrhea, and cough) as well as body temperature values were recorded throughout the experiment. Blood samples were obtained at every 5 days post inoculation (pi) and tested for the presence of ASFV using real time-PCR using VetMAX African Swine Fever Virus Detection Kit (Thermo Fisher Scientific, Cat # A28809, Waltham, MA, USA) as per the manufacturer’s instructions (Table 2, Figure 3).

**Figure 4:**
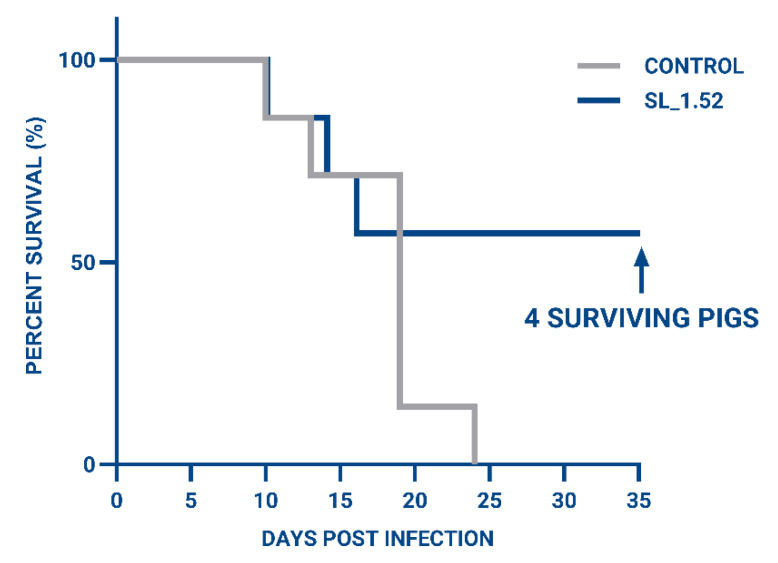
Overall survival of animals infected with a lethal dose of ASFV followed by the SL_1.52 treatment compared to control animals over a period of 35 days post treatment. Seven swine in each group were inoculated intramuscularly (IM) with 10^2^ HAD_50_ of ASFV/Thailand/2023, and treatment was administered at 5 dpi and 6 dpi.

It was observed that at 10 dpi, there was a statistically lower virus load in all of the treated animals, showing the effectiveness of SL_1.52 (Figure 3), and 4/7 animals receiving SL_1.52 (Pig#; SL_1.52-8, -10, -11, -12) survived beyond the untreated infected subjects (Figure 4) and were able to clear the virus to non-detectable levels in blood between 20 dpi and 35 dpi (Table 2). In SL_1.52-treated animal #7, a decrease in the amount of ASFV was also detected at 15 dpi. Interestingly, this animal had the lowest Ct value, indicating highest viral loads of all treated animals at this timepoint, and yet survived the longest of the non-surviving animals in the SL_1.52-treated group, succumbing to disease at 16 dpi. SL_1.52-treated animals #9 and #13 were euthanized at 10 dpi and 14 dpi, respectively. In the control untreated group, pigs #1 and #2 succumbed to acute ASFV at 10 dpi and 14 dpi, while pigs #3, #5, #6, and #7 succumbed to acute ASFV at 19 dpi and #4 at 25 dpi (Figure 4).

All surviving treated pigs were able to clear the virus and had undetectable viremia by 35 dpi (Table 2 No clinical signs were observed in either group until day 10, when one animal in both the treated and untreated groups succumbed to acute ASFV. Treated animals remained clinically normal for the remainder of the study. In contrast, untreated animals developed progressive ASFV-associated symptoms, including depression, anorexia, weakness, and cutaneous hemorrhages or erythematous lesions, that intensified over time. Animals exhibiting severe or prolonged clinical disease were humanely removed from the study, and in cases of rapid onset, intervention was initiated as promptly as possible, as indicated in Figure 4.

### 3.5. Evaluation of Immunity for Surviving Animals

**Figure 5:**
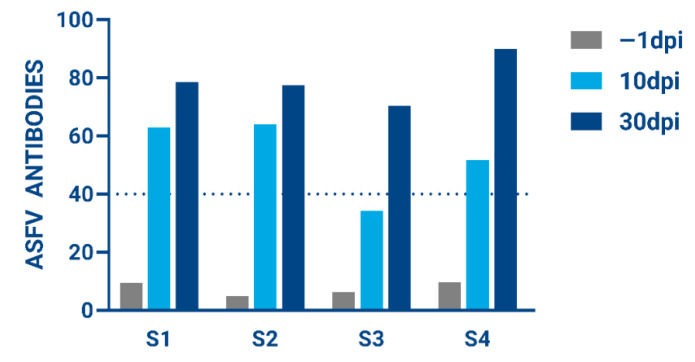
ASFV-specific antibodies were measured using the ID.Vet Elisa assay and calculated as a percentage of the positive control at −1, 10, and 30 dpi; 40% is considered a cutoff for positive ASFV antibodies.

Next, we wanted to determine if animals that survived the initial ASFV infection had developed immunity to protect against subsequent exposures to ASFV. Using an ASFV-specific antibody Elisa (ID.Vet), serum samples were tested against a positive antibody control, following manufacturer’s instructions. In all animals tested one day before ASFV inoculation, antibody levels were below the recommended detection threshold of 40%. However, all animals showed increased levels of ASFV-antibodies around 10 dpi and, by 30 dpi, all animals achieved antibody levels above the 40% threshold, indicative of a robust immune response (Figure 5).

**Figure 6:**
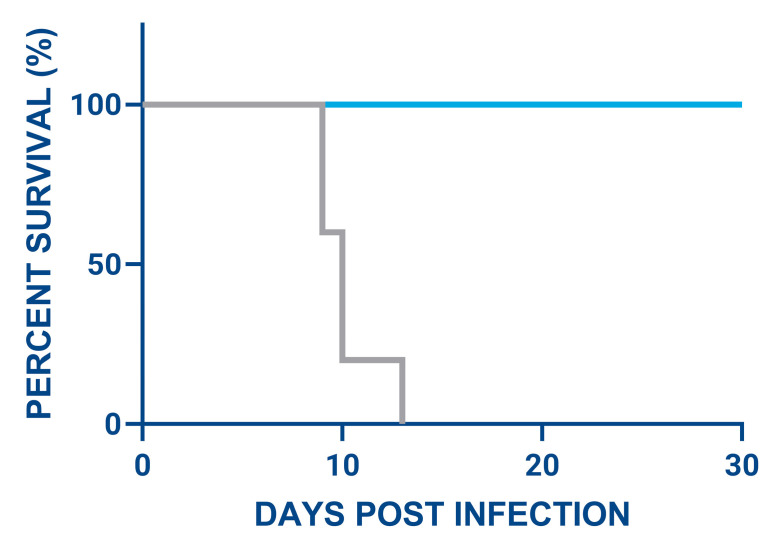
Evaluation of immunity to ASFV re-challenge in pigs surviving the first infection and treatment. In blue (*n* = 4), swine that survived the first ASFV challenge after SL_1.52 treatment in the prior study (Section 3.4); in grey are naïve control pigs (*n* = 5).

To determine if SL_1.52 treatment facilitated an immune response that was protective against future challenges, all surviving animals and control animals were given a second IM HAD 10^2^ lethal doses of ASFV/Thailand/2024 at 62 days post initial infection. All SL_1.52-treated animals that previously recovered from ASFV survived the second lethal ASFV challenge, suggesting that animals treated with SL_1.52 are protected from future ASFV infections. In contrast, all control animals succumbed to disease in the second study and had to be euthanized by 12 dpi (Figure 6).

## 4. Discussion

This study marks the first report that a CRISPR-based system is effective against ASF and could be used to allow infected swine to recover from a lethal infection of ASFV. With 57% of pigs surviving an otherwise lethal dose of ASFV, SL_1.52 was shown to be effective as a treatment. Surviving pigs had little to no clinical symptoms after treatment, and clearance of detectable levels of ASFV in the blood was observed by 35 dpi. As this was an initial proof-of-concept study, additional experimentation is necessary to further understand the mechanisms of why only some treated animals survived ASFV and how the use of different dosages, timing of treatment administration, or delivery strategies could improve the effectiveness of SL_1.52. It is also important to note that, in this study, all animals received a lethal dose of ASFV by intramuscular injection and that a contact challenge could have led to increased survival rates under conditions that are like a farm during an outbreak of ASFV. There are also several components that could be further optimized to increase the survival rate of ASFV-infected animals. Systems with additional guides that target more sites along the ASFV genome could improve disruption of viral replication. Furthermore, using novel LNP compositions to target biodistribution to different organs or tissues, including LNPs that could directly target tissue-resident macrophages that serve as viral reservoirs, may lead to better survival outcomes. However, this would require additional studies, including those to determine the effectiveness of SL_1.52 under field outbreak conditions.

Other research groups have tried to use CRISPR/Cas9 to target ASFV. In the report by Hubner et al. [33], researchers generated a stable cell line expressing a CRISPR/Cas9 targeting the p30 gene, which encodes the well-characterized capsid protein in ASFV. This approach rapidly induced selective pressure, causing mutations in the targeted nucleotide regions and the emergence of an escape mutant in the stable cell line. While it is interesting that ASFV could escape CRISPR treatment, this work was done under intense selective pressure in cell culture, and ASFV was likely undergoing adaptation to the WSL cell line, which could facilitate the generation of the identified escape mutants. Also, in this study, the authors used an NLS-deficient Cas9 [33], whereas in our study we used Cas9 containing an NLS to target initial ASFV replication in the nucleus with potential to be incorporated into viral factories as nuclear proteins are recruited to viral factories [34]. Another report by Zheng et al. [35] developed transgenic pigs expressing a CRISPR/Cas9 system with or without NLS with 9 different guides targeting ASFV genes. In this study, the NLS-containing Cas9 construct was more potent in cell culture and was selected to make transgenic pigs. While researchers observed no difference in pathogenesis between the control pigs and the genetically engineered pigs, genomic incorporation of ASFV targeting CRISPR/Cas9 system did limit transmission of ASFV to uninfected pigs [35]. It is possible that continuous expression of Cas9 and guides could be suppressed by the host cell or that exposure to the Cas9 system during early infection causes rapid selective pressure. Another possibility is that their transgenic Cas9 system did not express at sufficient levels in ASFV infected cells in the transgenic swine.

In our study, we were able to show that 4/7 CRISPR-treated swine survived a lethal dose of ASFV. Importantly, SL_1.52 is unlikely to create the selective pressure that was potentially observed in the other two studies. Since SL_1.52 is not present during the initial infection and ASFV is already present at detectable and often high levels before clinical symptoms are observed, the initial viremia observed would not have conferred selective pressure prior to our treatment with SL_1.52. It is feasible that SL_1.52 helps to decrease virus loads by cleaving ASFV DNA in treated cells, as was observed with decreased ASFV titers at 10 dpi in treated animals, reducing disease burden and allowing the pig’s natural immune system to mount an effective immune response, as indicated by high anti-ASFV antibody levels in surviving pigs. SL_1.52 also contains two distinct cleavage sites found in ASFV/Thailand/2023, shown to be effective for cleavage in vitro. Therefore, multiple mutation events would be required for an ASFV mutant to evolve and escape SL_1.52 treatment. Interestingly, SL_1.52-treated animals that succumbed to ASFV had a rapid onset of disease but were clinically normal the day before the onset of disease. This suggests that SL_1.52 was effective in reducing viral burdens as observed at 10 dpi, with 3 pigs showing rapid onset of disease at 10 dpi, 14 dpi, and 16 dpi.

Controlling ASFV outbreaks has been a problem for over a century; however, the disease has mostly remained endemic, with sporadic outbreaks being quickly resolved by aggressive swine depopulation strategies. The current spread of ASFV and continued outbreaks throughout Asia and Europe require additional rapidly deployed control measures such as anti-viral treatments. While commercial vaccines have started to be introduced in Vietnam, resistant strains have already been identified in these areas [15]. Here, we provide the potential for CRISPR/Cas to be deployed as a targeted treatment for ASFV, with the potential to combat multiple or all strains of ASFV. CRISPR/Cas systems can be reprogrammed with new or an expanded set of gRNA sequences, which allows for rapid adaptation to target different viral strains, multiple strains simultaneously, or emerging threats. Moreover, using such treatments in combination with vaccinations, where available, could control the disease more effectively, minimizing spread. A treatment for ASF like we present here can save infected swine from death or reduce the sale or trade of diseased animals by backyard- or small-scale farmers, thereby discouraging the spread of ASFV. Highly effective ASFV therapeutics could also potentially eliminate the need of drastic control measures, which are currently required, including massive culling of swine that occurs in new outbreak areas, mitigating devastating socioeconomic losses.

Programmable antiviral technologies, such as CRISPR, that can potentially be adapted within days to target new sequences could transform outbreak responses across multiple sectors. CRISPR-Cas9 has emerged as a promising antiviral modality that can function to disrupt infections in diverse ways. With the potential for new and re-emerging viral diseases or the increasing risk of bio-threats that endanger both human and animal health, the need for rapid and reliable responses are critical. Traditional vaccine and drug development pipelines depend on prior pathogen characterization, leaving significant gaps when new agents appear. CRISPR technology offers transformative potential to combat such threats by enabling rapid, precise destruction of viral genomes.

## Figures and Tables

**Table 1:** gRNAs designed in SL_1.52 targeting *G1211R*.

gRNA1	gattgttgcacgggagaacc
gRNA2	tttaacaatcgtctcgtgga

**Table 2:** Blood samples were taken every 5 days and virus load in pigs was determined by ASFV p72 gene expression, as measured by qPCR.

Animal	Days Post Infection								
	0	5	6	10	15	20	25	30	35
**Untreated-1**	N	34.16	28.00	22.68	-	-	-	-	-
**Untreated-2**	N	N	32.40	26.99	-	-	-	-	-
**Untreated-3**	N	N	23.84	29.16	34.00	-	-	-	-
**Untreated-4**	N	36.71	34.75	32.94	36.68	N	36.57	-	-
**Untreated-5**	N	36.22	36.75	31.30	30.98	-	-	-	-
**Untreated-6**	N	33.09	34.05	31.81	29.03	-	-	-	-
**Untreated-7**	N	41.42	N	30.94	34.44	-	-	-	-
**SL_1.52-8**	N	N	35.88	38.82	N	33.17	34.51	41.38	N
**SL_1.52-9**	N	N	N	41.89	-	-	-	-	-
**SL_1.52-10**	N	35.74	29.32	38.05	34.07	38.47	N	N	N
**SL_1.52-11**	N	40.18	36.34	37.96	28.84	N	N	N	N
**SL_1.52-12**	N	N	N	38.30	34.88	33.17	33.58	N	N
**SL_1.52-13**	N	38.44	38.59	36.51	-	-	-	-	-
**SL_1.52-14**	N	N	32.27	34.73	25.91	-	-	-	-

## Data Availability

All data is included in the manuscript.
